# Genetic diversity of *Toxoplasma gondii* isolates from members of the families camelidae and equidae worldwide: a systematic review

**DOI:** 10.1186/s13620-026-00344-4

**Published:** 2026-05-07

**Authors:** Maryam Hataminejad, Elham Kia Lashaki, Emad Behboudi, Behnaz Esmaeili, Somayyeh Ahmadi, Tahereh Mikaeili Galeh

**Affiliations:** 1 Student Research Committee, Mazandaran University of Medical Sciences, Sari, Iran; 2 Toxoplasmosis Research Center, Communicable Diseases Institute, Faculty of Medicine, Mazandaran University of Medical Sciences, Sari, Iran; 3 Department of Parasitology, School of Medicine, Mazandaran University of Medical Sciences, Sari, Iran; 4 Department of Parasitology and Mycology, To.C., Islamic Azad University, Tonekabon, Iran; 5 Department of Basic Medical Sciences, Khoy University of Medical Sciences, Khoy, Iran; 6 Department of Basic Medical Sciences, Faculty of Medicine, Maragheh University of Medical Sciences, Maragheh, Iran

**Keywords:** *Toxoplasma gondii*, Genetic diversity, Camelidae, Equidae, Systematic review, Genotyping

## Abstract

*Toxoplasma gondii* is an apicomplexan parasite with a global distribution and significant public health and veterinary treatment implications. The present systematic review collates worldwide information on the genotypic diversity of *T. gondii* isolates infecting Camelidae (camels, alpacas, llamas, etc.) and Equidae (horses, donkeys, zebras, mules, etc.) and highlights their potential role as sentinels of environmental contamination and zoonotic spread. An exhaustive literature search of six electronic databases (PubMed, Web of Science, Scopus, ProQuest, Science Direct, and Google Scholar) from inception to 20 July 2025 identified 20 relevant studies reporting genotypic information from 103 isolates. The present analysis reveals key geographical differences: atypical genotypes predominated in the Americas (88.2%) and Africa (83.3%), reflecting high regional diversity. Asian isolates featured the greatest complexity and presented mostly mixed infections (61.1%), and the China I genotype (ToxoDB #9) emerged as the dominant individual lineage. European isolates presented a very separate profile by mainly consisting of Type III (57.1%), but atypical genotypes were also reported. These findings indicate that Camelidae and Equidae hosts carry rich and diverse populations of *T. gondii* that closely mirror local strains in their environment rather than classical clonal Types. The co-existence of genotypes linked with human virulence (e.g., Type I and China I) in these food animals highlights their significant risk as sources in zoonotic transmission. This review advises the adoption of high genotyping resolution and One Health approaches within subsequent surveillance exercises to effectively assess public health risk and illuminate transmission dynamics relevant to these economically and culturally significant hosts.

## Introduction

Toxoplasmosis, induced by the obligate intracellular protozoan *Toxoplasma gondii*, is a worldwide distributed zoonosis that infects approximately one-third of the world’s human population [1, 2]. The disease imposes a substantive public health and economic impact, and infection arises by ingestion of tissue cysts in improperly cooked meat, ingestion of sporolated oocysts shed by cats, or by congenital transmission [2, 3]. Toxoplasmosis is asymptomatic in immunocompetent hosts, but can inflict severe clinical manifestations in congenitally infected fetuses and immunocompromised individuals [4, 5].

Clinical presentation and epidemiology of toxoplasmosis are shaped by a pluralistic interrelation between the immune status of the host and the genetic constitution of the parasite [6]. *T. gondii* has remarkable genetic complexity, and its population structure has been reported to be diverse across geographical regions [7, 8]. The genetic polymorphism is of paramount importance since various genotypes have been reported to be linked with variations in virulence, transmission modality, and outcome in human and animal hosts [9, 10]. Molecular methods, particularly the polymerase chain reaction-restriction fragment length polymorphism (PCR-RFLP) of various genetic loci and sequencing methodologies, are routinely adopted for genotyping the genetic polymorphism [11].

Apart from the human health significance, toxoplasmosis has great economic consequences within domestic animals due to abortions, stillbirths, and neonatal death [12]. Remarkably, a majority of research emphasis has mainly been on domestic species such as cattle, sheep, and goats; on the other hand, important species with economic, cultural, and environmental significance, such as those within the Camelidae (e.g., camels, llamas, and alpacas) and Equidae (e.g., horses, donkeys, and zebras) families, have been relatively ignored. Camel herds are core to semi-arid pastoral economies as a provider of meat and milk and for use as a beast of burden and a symbol with cultural significance [13, 14]. Likewise, species within the Equidae family are crucial for agriculture-related activities, meat production, racing, travel and therapy [15]. The two families are at risk for exposure to *T. gondii* due to environmental contact with oocysts [16]; however, their involvement in the epidemiology and strain make-up of the parasite is unidentified. As important food-producing animals with close contact with human communities, they are a potential zoonotic reservoir; therefore, the research on the epidemiology and genotyping of *T. gondii* in the animals constitutes a priority on veterinary and human health [17].

While various studies have documented seroprevalence and genetical profiling of *T. gondii* in camels and equids within various regions, a comprehensive global population structure of the parasite within the hosts has never been provided. Consequently, the current systematic review aspires to summarize and analyze accessible information on the genetic variation of *T. gondii* isolates in Camelidae and Equidae globally.

## Materials and methods

### Research design

The systematic review proposed to comprehensively map the genetic variation for *T. gondii* isolates from Camelidae and Equidae worldwide. This work was carried out in accordance with the items reported in the preferred reporting items for systematic reviews (PRISMA) statement [18].

### Literature search strategy

To collect all available evidence, systematic search was also conducted on six electronic databases: PubMed, Web of Science, Scopus, ProQuest, Science Direct, and Google Scholar, from their inception to 20 July 2025. The search strategy covered both controlled vocabulary (e.g., MeSH terms) and keywords that covered three areas: target hosts (“camels”, “llamas”, “alpacas”, “horses”, “donkeys”, “mules”, “zebras”, etc.), pathogens (“*Toxoplasma gondii*” and “toxoplasmosis”), and terms related to genotyping (“genotype”, “genotyping”, “genetic characterization”, “genetic diversity”, “multilocus sequence typing”, “MLST”, “PCR-RFLP”, and “sequence analysis”). All these terms were linked by Boolean operators (AND, OR) and were tailored for each database’s specific query dialect. Reference lists of included studies were hand-screened for augmentation of databases search. There was no restriction on date of publication.

### Eligibility for studies

Surveys were eligible for inclusion if they presented primary genetic data on *T. gondii* isolates from Camelidae or Equidae hosts, used traditional molecular characterization methods, and provided original experimental data. Reviews, editorials, non-English language papers and studies that lacked references to the host species or isolate origins were ruled out.

### Procedure for screening and selecting

Two consecutive phases were involved in the screening. Two autonomous reviewers initially scanned titles and abstracts of all records identified for exclusion of obviously irrelevant studies. Thereafter, full texts of possibly eligible papers were vigorously tested against inclusion criteria. Disputes among reviewers were addressed by consensus discussions or by a third reviewer’s arbitration. The complete selection workflow, including reasons for exclusion, was presented in a PRISMA flow diagram.

### Data extraction strategy

Data were extracted from the included studies using a standardized, pilot-tested form. Data extraction focused on four key domains: study context, laboratory methodology, strain characteristics, and epidemiological variables. The extracted data were systematically organized into an evidence table with the following headings: Continent, References, Sample(s), Country, Diagnostic techniques, Molecular marker(s), Host, No. DNA or isolates, Type(s) No. (%) or Genotype(s) No. (%), No. genotypes, and ToxoDB Genotype (No.) to facilitate a comprehensive analysis of the findings. ToxoDB genotypes were determined by comparing and matching to the known reference RFLP genotypes listed in the ToxoDB genotyping database (www.toxodb.org).

### Phylogenetic analysis

SplitsTree4 software (version 4.17.2) was used to create a phylogenetic network using the neighbor-joining algorithm and distances were computed using the Tajima-Nei method. Isolates genotyped with at least 11 multi-locus RFLP markers were chosen for the analysis. Finally, data obtained by PCR-RFLP method (alleles I, II, III, II/III, u-1, and u-2) was transformed into binary data and tabulated.

## Results

### Study selection and characteristics

**Fig. 1:**
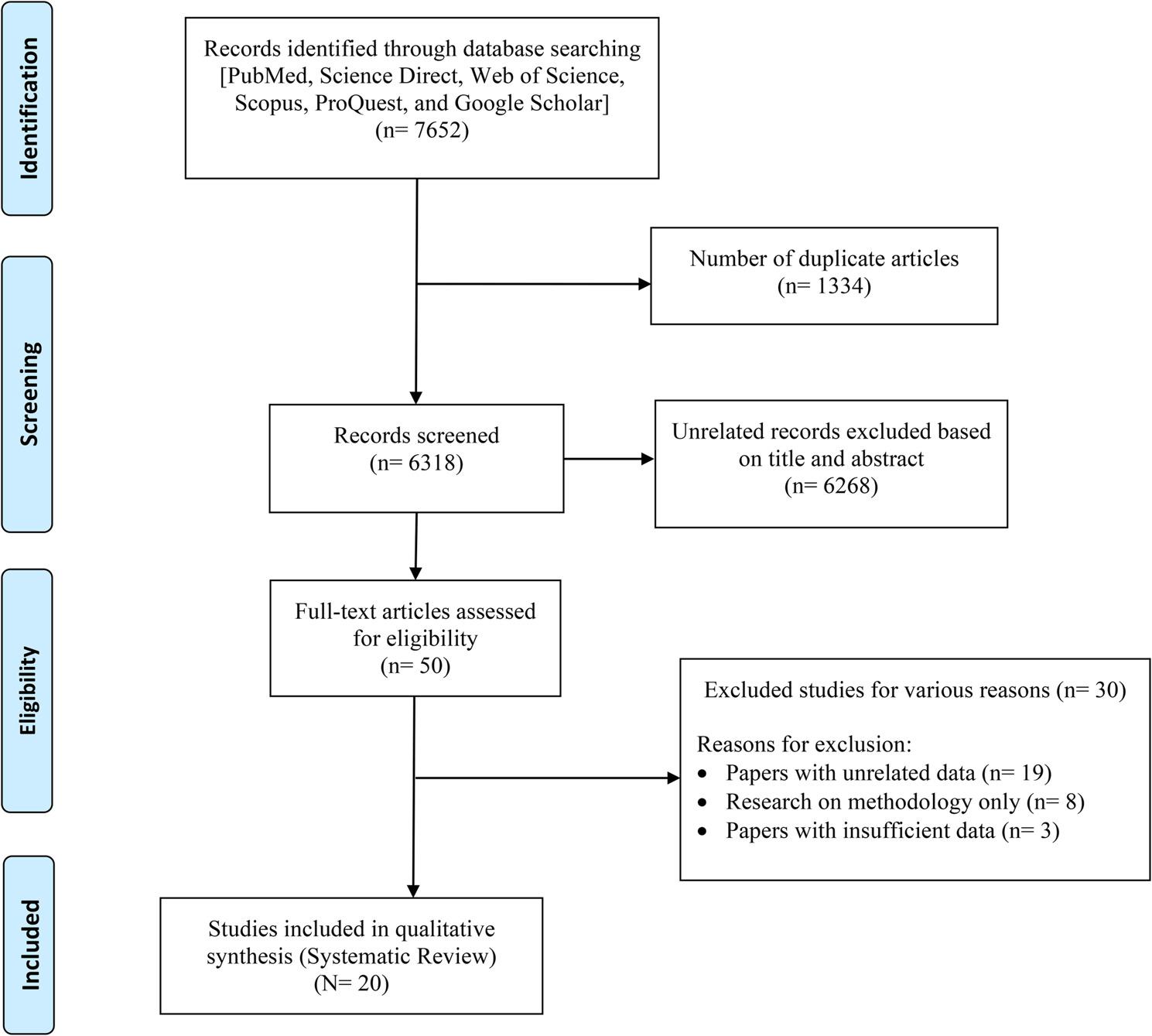
PRISMA flow diagram describing included and excluded studies

**Fig. 2:**
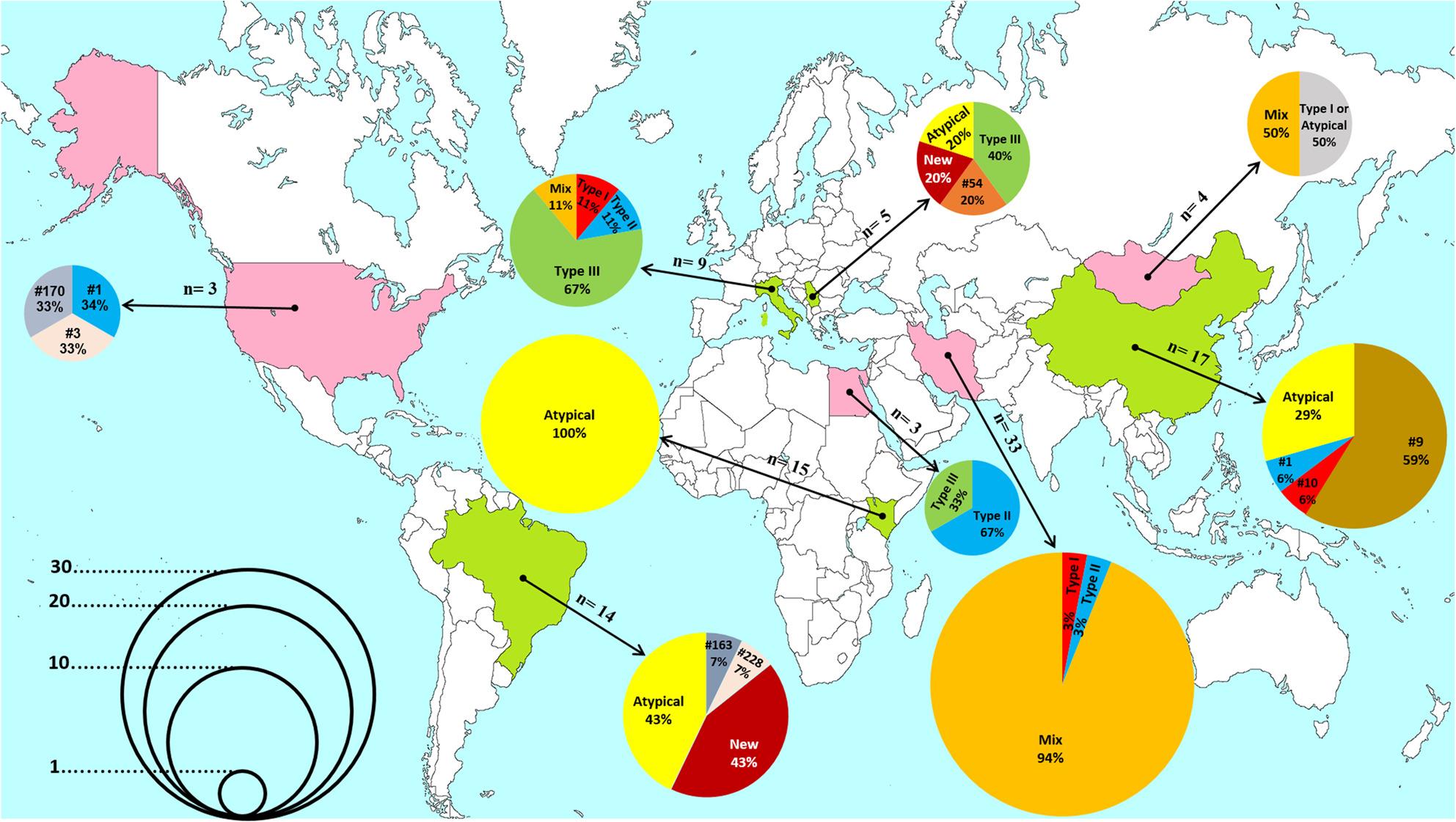
Geographical distribution of T. gondii genotypes from the members Camelidae and Equidae families in the world. Sizes of pie charts correlate with the total number of isolates (*n*) reported for each country. Pink and green colors indicate locations with data for the members Camelidae and Equidae families, respectively. White colors indicate locations without data

A systematic search of six electronic databases (PubMed, Web of Science, Scopus, ProQuest, Science Direct, and Google Scholar) from inception through 20 July 2025 yielded a total of 7,652 records. After removal of duplicates and a rigorous two-stage screening process based on title, abstract, and subsequently full-text assessment, 20 studies met the predefined eligibility criteria and were included in this systematic review. The study selection process, detailed in the PRISMA flow diagram (Fig. 1), ensured the inclusion of all relevant English-language articles published from 1992 onwards. The geographical scope of the included studies was global, with a thematic focus on the genetic characterization of *T. gondii* in members of the Equidae (*n* = 12 studies) and Camelidae (*n* = 8 studies) families. The distribution of these studies across countries and continents is summarized in Table 1, providing the foundation for the subsequent analysis of genetic diversity [19–38]. Figure 2 displays the geographic distribution of *T. gondii* genotypes in the members Camelidae and Equidae families worldwide.

**Table 1:** Basic characteristics of selected studies and genetic characterization of *T. gondii* isolates from members of the families Equidae and Camelidae worldwide

Continent/ References	Sample(s)	Country	Diagnostic techniques	Molecular marker(s)	Host	No. DNA or isolates	Type(s) No. (%) or Genotype(s) No. (%)	No. genotypes	Toxo DB Genotype (No.)
Equidae									
Evers et al-2013 [20]	Sera and Brain	Brazil	PCR-RFLP	SAG1, alt-SAG2, 5’-3’SAG2, SAG3, BTUB, GRA6, C22-8, C29-2, L358, PK1, Apico, and CS3	Horse	3	Atypical: 3 (100)*		
Mancianti et al-2014 [23]	Milk and Blood	Italy	PCR-RFLP	SAG1, 5’-3’SAG2, alt-SAG2, SAG3, BTUB, GRA6, C22-8, C29-2, L358, PK1, and Apico	Donkey	6	Type II: 1 (16.7) and Type III: 5 (83.3)*		
Gennari et al-2015 [21]	Brain, Tongue, Diaphragm, and Heart	Brazil	PCR-RFLP	SAG1, 5’-3’SAG2, alt-SAG2, SAG3, BTUB, GRA6, C22-8, C29-2, L358, PK1, Apico and CS3	Donkey	1	Atypical: 1 (100)	1	#163 (1)
Papini et al-2015 [24]	Tongue, Masseter muscle, and Heart	Italy	PCR-RFLP	SAG1, 5’-3’SAG2, alt-SAG2, SAG3, BTUB, GRA6, C22-8, C29-2, L358, PK1, and Apico	Horse	3	Type I: 1 (33.3), Type III: 1 (33.3), and Mix: 1 (33.4)*		
Zhang et al-2017 [28]	Brain	China	PCR-RFLP	SAG1, 5’-3’SAG2, alt-SAG2, SAG3, BTUB, GRA6, C22-8, C29-2, L358, PK1, and Apico	Donkey	5	Type I: 1 (20) and Atypical [China 1]: 4 (80)	2	#9 (4) and #10 (1)
Klun et al-2017 [22]	Sera, Brain, and Heart	Serbia	Multiplex PCR (microsatellite markers)	TUB2, W35, TgM-A, B18, B17, M33, IV.1, X1.1, N60, N82, AA, N61, N83, M48, and M102	Horse	2	Type III: 2 (100)		
Cong et al-2018 [19]	Muscle tissue	China	PCR-RFLP	SAG1, 5’-3’SAG2, alt-SAG2, SAG3, BTUB, GRA6, C22-8, C29-2, L358, PK1, and Apico	Donkey	5	Type II clonal: 1 (20) and Atypical [China 1]: 4 (80)	2	#9 (4) and #1 (1)
Pena et al-2018 [25]	Blood, Heart, and Brain	Brazil	PCR-RFLP and Multiplex PCR (microsatellite markers)	SAG1, 5’-3’SAG2, alt- SAG2, SAG3, BTUB, GRA6, C22-8, C29-2, L358, PK1, and Apico/TUB2, W35, TgM-A, B18, B17, M33, IV.I, X1.1, M48, M102, N60, N82, AA, N61, and N83	Horse	1	Atypical: 1 (100)	1	#228 (1)
Ren et al-2019 [26]	Cervical lymph node	China	PCR-RFLP	SAG1, alt-SAG2, SAG3, BTUB, GRA6, C22-8, C29-2, PK1, and an Apico	Horse	7	Atypical: 7 (100)*	1	#9 (2)
Uzelac et al-2021 [27]	Heart	Serbia	PCR-RFLP	alt-SAG2, GRA6, BTUB, C22-8, C29-2, L358, PK1, Apico, and CS3	Horse	3	Atypical: 3 (100)*	2	#54 (1) and New (1)
Obonyo et al-2024 [37]	Blood samples	Kenya	PCR-RFLP and Sequencing	alt-SAG2, 5’-3’SAG2, SAG3, BTUB, GRA6, C29-2, and L358	Donkey	15	Atypical: 15 (100)		
de Oliveira et al-2025 [38]	Masseter muscles and Brains	Brazil	PCR-RFLP and Sequencing	SAG1, 5’-3’SAG2, alt-SAG2, SAG3, BTUB, GRA6, C22-8, C29-2, L358, PK1, Apico, and CS3	Horse	9	Atypical: 9 (100)	6	New (6)
Camelidae									
Dubey et al-2014 [31]	Muscles (heart and skeletal muscle)	USA	PCR-RFLP	SAG1, 5’-3’SAG2, alt-SAG2, SAG3, BTUB, GRA6, C22-8, C29-2, L358, PK1, and Apico	Alpaca	2	Type II variant: 1 (50) and Atypical: 1 (50)	2	#170 (1) and #3 (1)
Dubey et al-2014 [32]	Liver, Kidney, Spleen, Heart, Diaphragm, Thyroid, Adrenal gland, C3 stomach, Small intestine, Cerebrum, Cerebellum, and Pons	USA	PCR-RFLP	SAG1, SAG2, SAG3, BTUB, GRA6, C22-8, C29-2, L358, PK1, and Apico	Llama	1	Type II clonal: 1 (100)	1	#1 (1)
Aliabadi et al-2016 [29]	Diaphragm and Heart	Iran	PCR-RFLP	B1	Camel	19	Mix: 19 (100)		
Elfadaly et al-2017 [34]	Blood and Diaphragm tissue	Egypt	PCR-RFLP	5’-3’SAG2	Camel	2	Type II: 1 (50) and Type III: 1 (50)		
Tavakoli kareshk et al-2018 [36]	Diaphragm and Heart	Iran	PCR-RFLP and Sequencing	GRA6	Camel	13	Type II: 1 (7.7) and Mix: 12 (92.3)		
El-Alfy et al-2019 [33]	Fecal	Egypt	PCR-RFLP and Sequencing	5’-3’SAG2 and alt-SAG2	Camel	1	Type II: 1 (100)		
Iacobucci et al-2019 [35]	Milk	Mongolia	Sequencing	B1	Camel	4	Mix: 2 (50) and Type I or Atypical: 2 (50)		
Azimpour-Ardakan et al-2021 [30]	Muscle tissue	Iran	Sequencing	B1 and ROP8	Camel	1	Type I: 1 (100)		

* All or some of the isolates were not amplified in all of the markers used

### Continental distribution and genetic characterization of *T. gondii* isolates

The 20 eligible studies provided a global overview of *T. gondii* genetic diversity in camelids and equids, encompassing isolates from five continents: North America, South America, Asia, Europe, and Africa. A total of 103 isolates were genetically characterized. The distribution of these isolates, along with their predominant Types and ToxoDB PCR-RFLP genotypes, is detailed below and summarized in Tables 2 and 3.

**Table 2:** Genetic characterization of *T. gondii* isolates from members of the families Equidae and Camelidae worldwide by continent and country, and by family

Continent/Country	No. isolates	Type I Type II Type III Atypical Mix No. (%) No. (%) No. (%) No. (%) No. (%)	ToxoDB genotype (No.)
Asia			
Equidae			
China	17	1 (5.9) 1 (5.9) - 15 (88.2) -	#1 (1), #10 (1), #9 (10)
South America			
Brazil	14	- - - 14 (100)*-	#163 (1), #228 (1), New (6)
Europe			
Serbia	5	- - 2 (40) 3 (60)*-	#54 (1), New (1)
Italy	9	1 (11.1 ( 1 (11.1) 6 (66.7)* - 1 (11.1)*	
Africa			
Kenya	15	- - - 15 (100) -	
	**Total: 60**	**2 (3.3) 2 (3.3) 8 (13.3) 47 (78.4) 1 (1.7)**	**#1 (1)**,** #10 (1)**,** #9 (10)**,** #54 (1)**,** #163 (1)**,** #228 (1)**,** New (7)** **Total: 22**
Camelidae			
Asia			
Iran	33	1 (3) 1 (3) - - 31 (94)	
Mongolia	4	2 (50)** - - - 2 (50)	
North America			
USA	3	- 2 (66.7) - 1 (33.3) -	#1 (1), #3 (1), #170 (1)
Africa			
Egypt	3	- 2 (66.7) 1 (33.3) - -	
	**Total: 43**	**3 (7) 5 (11.6) 1 (2.3) 1 (2.3) 33 (76.8)**	**#1 (1)**,** #3 (1)**,** #170 (1)** **Total: 3**

* All or some of the isolates were not amplified in all of the markers used

** Type I or Atypical

**Table 3:** Genetic characterization of *T. gondii* isolates from members of the families Equidae and Camelidae worldwide by continent and country

Continent/Country	No. isolates	Type I Type II Type III Atypical Mix No. (%) No. (%) No. (%) No. (%) No. (%)	ToxoDB genotype (No.)
Equidae and Camelidae			
Asia			
China	17	1 (5.9) 1 (5.9) - 15 (88.2) -	#1 (1), #10 (1), #9 (10)
Iran	33	1 (3) 1 (3) - - 31 (94)	
Mongolia	4	2 (50)** - - - 2 (50)	
	**Total:54**	**4 (7.4) 2 (3.7) - 15 (27.8) 33 (61.1)**	**#1 (1)**,** #10 (1)**,** #9 (10)**
Americas			
Brazil	14	- - - 14 (100)*-	#163 (1), #228 (1), New (6)
USA	3	- 2 (66.7) - 1 (33.3) -	#1 (1), #3 (1), #170 (1)
	**Total:17**	**- 2 (11.8) - 15 (88.2) -**	**#1 (1)**,** #3 (1)**,** #163 (1)**,** #170 (1)**,** #228 (1)**,** New (6)**
Europe			
Serbia	5	- - 2 (40) 3 (60)*-	#54 (1), New (1)
Italy	9	1 (11.1 ( 1 (11.1) 6 (66.7)* - 1 (11.1)*	
	**Total:14**	**1 (7.2) 1 (7.2) 8 (57.1) 3 (21.4) 1 (7.1)**	**#54 (1)**,** New (1)**
Africa			
Kenya	15	- - - 15 (100) -	
Egypt	3	- 2 (66.7) 1 (33.3) - -	
	**Total:18**	- **2 (11.1) 1 (5.6) 15 (83.3) -**	

* All or some of the isolates were not amplified in all of the markers used

** Type I or Atypical

### Americas

Data from the Americas were derived from two studies in the United States (camelids) and four studies in Brazil (equids), yielding 17 isolates for analysis. Genetic characterization revealed a complete absence of the classic Types I and III clonal lineages. The majority of isolates (88.2%, *n* = 15) were classified as atypical, demonstrating a high degree of genetic diversity in this region. The remaining isolates (11.8%, *n* = 2) belonged to the Type II clonal lineage. The following ToxoDB genotypes were identified by PCR-RFLP analysis: #1 (Type II clonal), #3 (Type II variant), #163, #170, #228, and a few newly reported genotypes.

### Asia

Asia was the most represented continent with seven studies: three from China (equids), three from Iran (camelids), and one from Mongolia (camelids). From these, 54 isolates were genotyped. This continent exhibited the greatest genetic complexity. Mixed genotypes, indicating exposure to multiple *T. gondii* strains, were the most prevalent finding (61.1%, *n* = 33). Among single genotypes, atypical strains were prominent (27.8%, *n* = 15), more than half of which were identified as the China I genotype. The classic clonal Types were less common, with Type I at 7.4% (*n* = 4) and Type II at 3.7% (*n* = 2). ToxoDB genotyping confirmed the prevalence of #9 (China I) genotype. Genotypes #1 and #10 (Type I clonal) were also reported, solidifying the conclusion that the China I genotype is the most prevalent single genotype in Asia.

### Europe

Four studies from Europe (two each from Serbia and Italy, on equids) contributed 14 isolates. The genotyping profile in Europe was distinct, with Type III being the predominant clonal lineage (57.1%, *n* = 8). Types I and II were each identified in a single isolate (7.2% each). Atypical strains accounted for 21.4% of isolates (*n* = 3), and one mixed infection (7.1%) was reported. ToxoDB genotyping confirmed the presence of genotype #54 along with a novel genotype.

### Africa

Data from Africa were limited to three studies: two from Egypt (camelids) and one from Kenya (equids), from which 18 isolates were characterized. Similar to the Americas, no Type I strains were detected. The vast majority of isolates were atypical (83.3%, *n* = 15), underscoring the high genetic diversity of *T. gondii* in African camelids and equids. Type II and Type III lineages were minor components, representing 11.1% (*n* = 2) and 5.6% (*n* = 1) of isolates, respectively. Notably, no specific ToxoDB genotypes were definitively identified from the isolates in this region based on the available data.

### Phylogenetic analysis

**Fig. 3:**
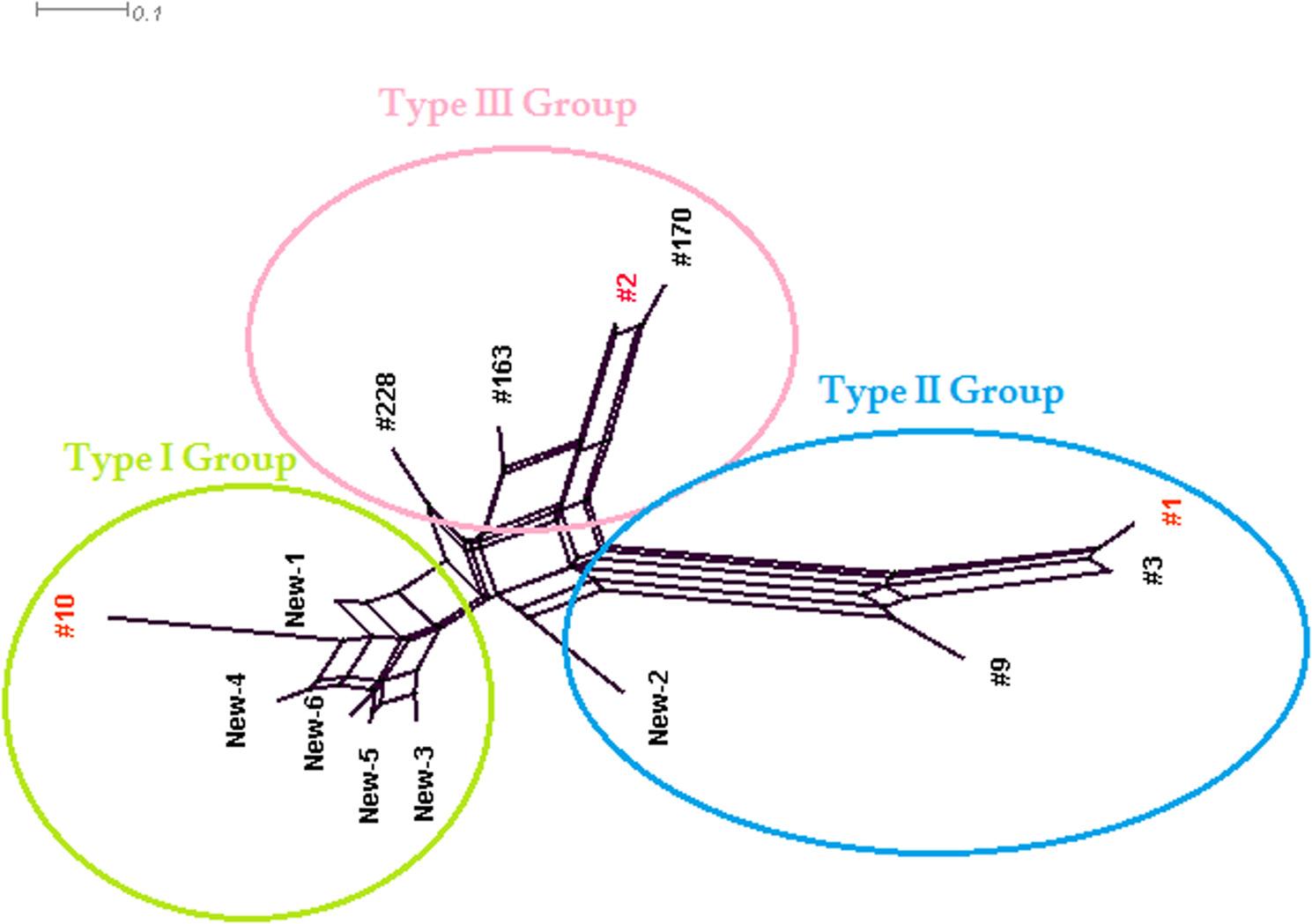
Phylogenetic network analysis of T. gondii isolates from the members Camelidae and Equidae families in the world. The neighbor-net analysis of 14 genotypes was conducted using at least 11 multilocus RFLP markers and the isolates fall into three distinct genetic groups in the phylogenetic tree

By drawing a phylogenetic tree using 14 isolates from the members Camelidae and Equidae families worldwide, *T. gondii* genotypes were classified into three distinct groups: Group (1) Type I strains or genotypes that are closely related to this lineage. Group (2) Type II strains or genotypes that are closely related to this lineage. Group (3) Type III strains or genotypes that are closely related to this lineage (Fig. 3). The genotypes in each of the three groups are related to the mentioned reference genotypes and likely are derived from a common ancestor.

## Discussion

This systematic review represents the first comprehensive analysis of the genetic diversity and population structure of *T. gondii* in members of the families Camelidae and Equidae on a global scale. By consolidating data from 20 studies, we have delineated a complex and geographically heterogeneous population structure of *T. gondii* in these economically and culturally significant animals. The central finding of this review is the overwhelming predominance of atypical or non-clonal genotypes across all continents, accounting for the majority (46.6%, 48/103) of characterized isolates. This highlights a genetic landscape far more diverse than the traditional clonal paradigm (Types I, II, and III) and underscores the unique epidemiological roles these host species may play.

The population structure we observed in camelids and equids demonstrates a clear pattern when compared to other host species. The overwhelming dominance of atypical genotypes aligns most closely with the genetic profiles found in dogs (78.2% atypical) [39], rodents (65.2% atypical), and birds (58.8% atypical) [40, 41]. This triad of hosts (dogs, birds, and large herbivores like camels and horses) share a common trait: they are primarily infected through environmental exposure to oocysts shed by felids. Dogs, through their coprophagic and scavenging behavior, and birds, through ground-feeding, are established bio-indicators of soil contamination [42–45]. Our findings strongly suggest that camelids and equids, due to their grazing habits, belong to this same category. The genetic diversity of *T. gondii* that they harbor is a direct reflection of the genetic diversity of oocysts in their ecosystem. This stands in stark contrast to the population structure in ruminants (81.4% Type II) [46] and humans (58% Type II) [47], where Type II clonal dominates. This discrepancy is likely driven by differing transmission routes. Humans are often infected through the consumption of tissue cysts in undercooked meat, creating a bottleneck that favors the propagation of a few successful, widely disseminated clonal lineages adapted to chronic infection. The pattern in cats (47.7% atypical, 37% Type II) [48] is intriguing, as the definitive host is exposed to both oocysts (from other cats) and tissue cysts (from prey), resulting in an intermediate level of genetic diversity. The high frequency of mixed infections in Asian equids and camelids (61.1%) is a particularly significant finding. It indicates a high intensity of environmental contamination and simultaneous exposure to multiple strains, a phenomenon also noted in Australasian marsupials [49]. This has profound implications for parasite recombination and the emergence of new genotypes, potentially influencing virulence and transmission patterns.

The high prevalence of atypical genotypes in camelids and equids is a cornerstone of our findings. This pattern is not isolated but rather mirrors the predominant population structure described across South America and Africa. The review by Brito et al. [50] emphatically confirms that South America is a hotspot for “non-archetypal (atypical) strains, with highly diverse genotypes [50]. Our data, showing 88.2% atypical strains in the Americas (with strong representation from Brazil), perfectly align with this continental characterization, suggesting camelids and equids are excellent indicators of this regional genetic richness of *T. gondii*. Similarly, the profile we identified in Africa (83.3% atypical genotypes) is corroborated by du Plooy et al. [51]. Their work describes a relative amount of genetic diversity in Africa, with a propensity for unique and regional genotypes to be predominant [51]. The scarcity of classic Type I and low frequency of Types II and III in African camelids and equids, as we found, fits within this framework of a unique and diverse African *T. gondii* population structure, likely influenced by tropical rainforest biomes and distinct transmission cycles. Asia presented the most complex picture in our review, with mixed infections being the most common finding. This indicates a high intensity of environmental contamination and co-infection events. The prominence of the #9 (China I) genotype aligns perfectly with the continental analysis by Chaichan et al. [52], which identified ToxoDB #9 as the most common genotype found in Asia [52]. Our review strengthens this by demonstrating the China I genotype’s significant presence not just in wildlife or cats, but also in economically important equids and camelids. This establishes a potential transmission link between these livestock, the environment, and human populations, where this genotype has also been implicated in clinical disease. Furthermore, the presence of Type I and Type II strains in Asia, though less common, reflects the continuum with Europe in Western Asia and the complex circulation of strains through trade and animal migration, as suggested by Chaichan et al. [52]. The European scenario in our data was distinct, with a clear dominance of the Type III clonal lineage (57.1%) in equids. This finding adds a critical layer to the European perspective. While the review by Uzelac et al. [53] discusses the tremendous increase in knowledge of the parasite’s genetic diversity and the development of sophisticated genotyping tools in Europe, it also acknowledges that early genotyping was heavily focused on humans and food animals, where Type II is often dominant [53]. Our results suggest that the population structure in equids may differ, highlighting a previously underappreciated reservoir of Type III. However, the concurrent presence of atypical strains (21.4%) and a new genotype in our data supports Uzelac et al.‘s conclusion that high-resolution genotyping is revealing greater diversity than previously assumed, even within the European context.

The genetic diversity of *T. gondii* evidenced by camelids and equids is more likely indicative of environmental contamination with a variety of genetically distinct oocysts than a clearly defined cycle of transmission. Unlike rodents, which are prey to cats and therefore complete the parasite’s life cycle [41], infection in camelids and equids is for all intents a dead-end for the parasite [15, 54] unless their parasite-infected carcasses are directly accessible to cats or other hosts that are ultimately preyed upon by cats. The strains they harbor are therefore direct results of exposure to the environment. This makes them valuable sentinels for mapping the genetic variability of *T. gondii* in a geographic area. The significant variability that has been reported here suggests that the common environments of these animals, other livestock, and human hosts are reservoirs of genetically complex *T. gondii* populations. The sentinel role of equids and camelids is of direct zoonotic significance. As food animals in the majority of cultures, they offer a direct route of transmission for these disperse strains to the human host. This represents a potential pathway: from the environment (oocysts) to sentinels (camelids, equids, birds, etc.) and to human hosts through the ingestion of improperly cooked meat or milk, etc. Therefore, genotyping in such hosts is not just intellectual exercise but necessary for proactive public health risk assessment and monitoring.

This review is subject to limitations, primarily due to the limited number of published studies and isolates particularly from under represented regions such as Southeast Asia, and Australia, that can possibly provide it geographic and taxonomic bias. In addition, methodological heterogeneity must be acknowledged: while previous studies relied on PCR-RFLP techniques capable of identifying major clonal Types, it undoubtedly underestimated the overall magnitude of genetic diversity, such as mixed infections and recombinant strains, while more high-resolution tools like microsatellite typing and whole-genome sequencing (WGS) can tease apart. Future studies need to involve enhanced monitoring in overlooked regions, high-density genotyping to validate population structure and virulence trait markers, and efforts to collect viable parasites for experimental models to test pathogenicity.

## Conclusion

This systematic review establishes that members of the families Camelidae and Equidae are infected by a vast array of *T. gondii* genotypes, with a strong global predominance of atypical and mix genotypes. This genetic richness firmly positions them as critical sentinels for environmental oocyst contamination and mirrors the biogeographic paradigms of the parasite, highlighting the Americas, Africa, and parts of Asia as hotspots of diversity, while Europe exhibits a more clonal structure with emerging variety. Most critically, the frequent presence of genotypes known to be virulent in humans (e.g., Type I and China I) in these animals, which are often consumed as food, elevates their significance from mere economic interest to vital reservoirs for zoonotic transmission. Therefore, sustained and sophisticated genetic surveillance of *T. gondii* in camelids and equids is an indispensable component of a comprehensive One Health strategy, essential for understanding and mitigating the global public health threat of toxoplasmosis.

## Data Availability

No datasets were generated or analysed during the current study.
